# Supplementary material for: A developmentally controlled cellular decompartmentalization process executes programmed cell death in the Arabidopsis root cap
Source: Plant Cell. Author manuscript; Available in PMC 2024 Sep 29. (PMC7615778; doi:10.1093/plcell/koad308)
Supplement: Figures [file EMS192759-supplement-Figures.docx]

**Supplemental Figure S1**

**
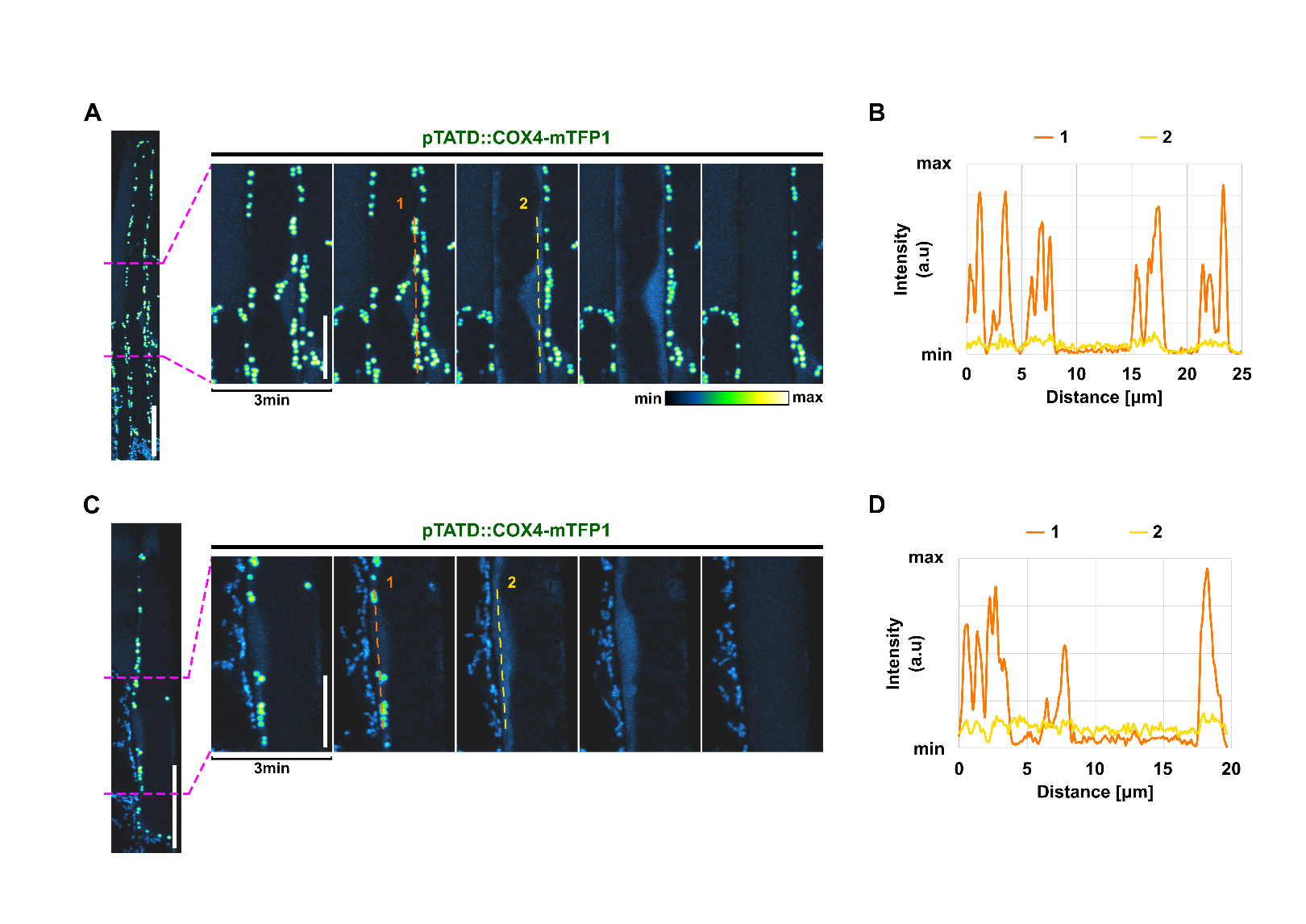
**

**Supplemental Figure S1:**  **Mitochondrial leakage into the cytosol precedes vacuolar collapse during PCD execution.** **(A, C)** Two representative time-lapse series of dying LRC cells expressing *pTATD::COX4-mTFP1* showing the leakage of mitochondrial contents into cytoplasmic space before vacuolar collapse. Dashed orange and yellow lines indicate region where signal intensity was profiled, which are plotted over distance in correlated graphs **(B, D)**. **Supports Figure 1**.


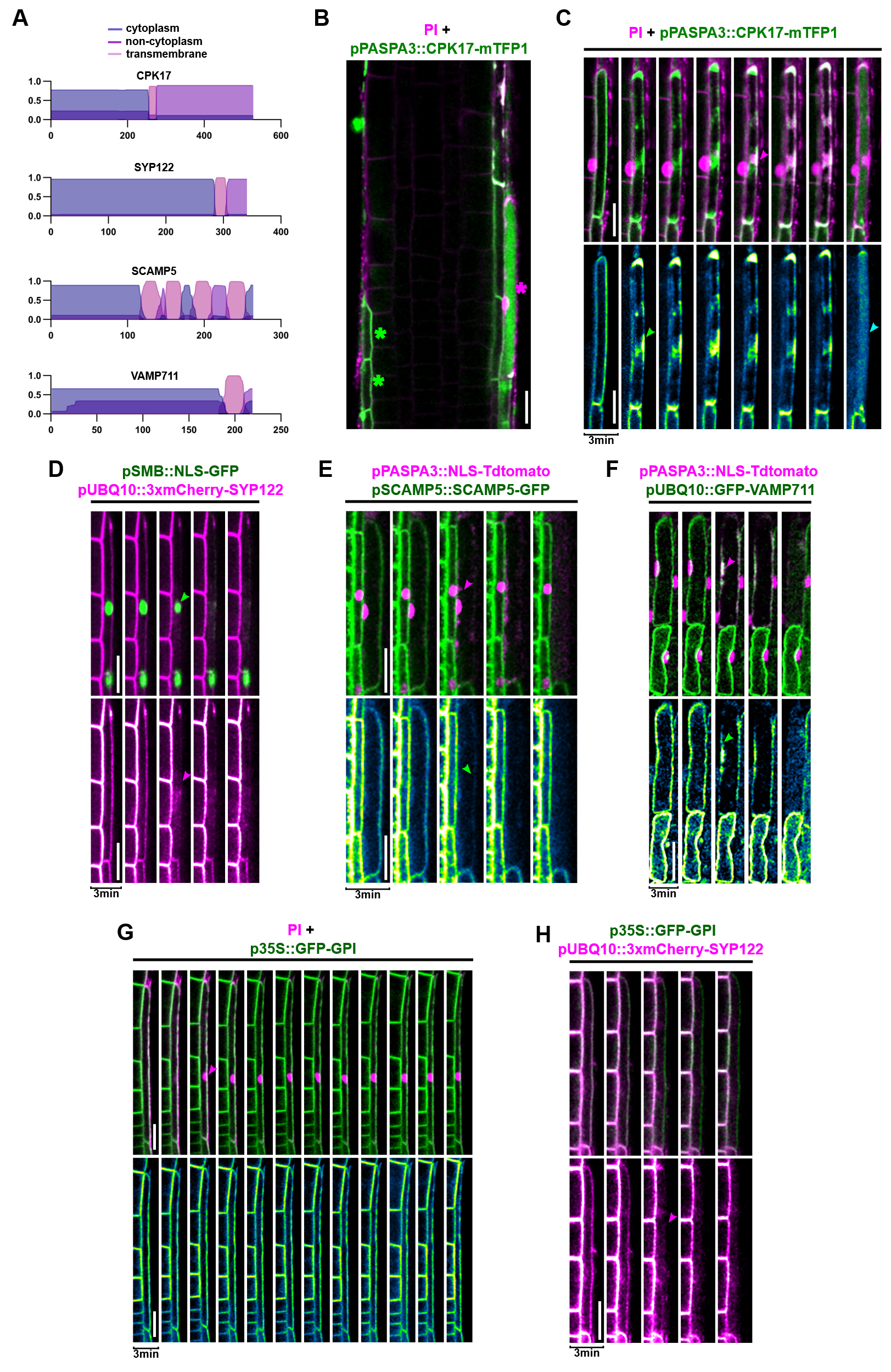
**Supplemental Figure S2**

**Supplemental Figure S2:** **Dynamic behaviors of plasma membrane- and tonoplast-localized proteins during PCD execution. (A)** Prediction of transmembrane domains in PM-localized (CPK17, SYP122 and SCAMP5) and tonoplast-localized (VAMP711) proteins. **(B)** Confocal images root cap cells expressing CPK17-mTFP1 in 4-day-old Arabidopsis seedlings. Green asterisks indicate living LRC cells with PM-localized CPK17-mTFP1, magenta asterisk indicate dying or dead cells. **(C)** Confocal time-lapse series of dying LRC cells. The upper panel shows PI in magenta and mTFP1 in green, the lower panel shows the mTFP1 channel separately. The green arrow indicates PM endodomain shedding, the magenta arrow indicates PI entry, and the cyan arrow indicates vacuolar collapse. **(D)** Confocal time-lapse series showing the simultaneous NE breakdown (green arrow) and PM shedding (magenta arrow) during PCD execution. The upper panel shows mCherry in magenta and NLS-GFP in green, the lower panel shows the mCherry channel separately. **(E)** Confocal time-lapse series showing the simultaneous NE breakdown (magenta arrow) and PM shedding (green arrow) during PCD execution. The upper panel shows NLS-TdTOMATO in magenta and SCAMP5-GFP in green, the lower panel shows the GFP channel separately. **(F)** Confocal time-lapse series showing the simultaneous NE breakdown (magenta arrow) and solubilization of the vacuole membrane marker GFP-VAMP711 (green arrow) during PCD execution. The upper panel shows NLS-TdTOMATO in magenta and GFP-VAMP711 in green, the lower panel shows the GFP channel separately. **(G)** Confocal time-lapse series showing PI entry (magenta arrow) and maintenance of apoplastic GFP-GPI after cell death execution. The upper panel shows PI in magenta and GFP-GPI in green, the lower panel shows the GFP channel separately. **(H)** Confocal time-lapse series of a dual marker line of mCherry-SYP122 (magenta, fluorescent tag on the cytoplasmic side) and GFP-GPI (green, fluorescent tag on the apoplastic side) showing the solubilization of mCherry in the cytoplasm (magenta arrow), while the extracellular GFP-GPI remains PM-bound. Scale bars are 20 μm. **Supports Figure 2**.

**Supplemental Figure S3**


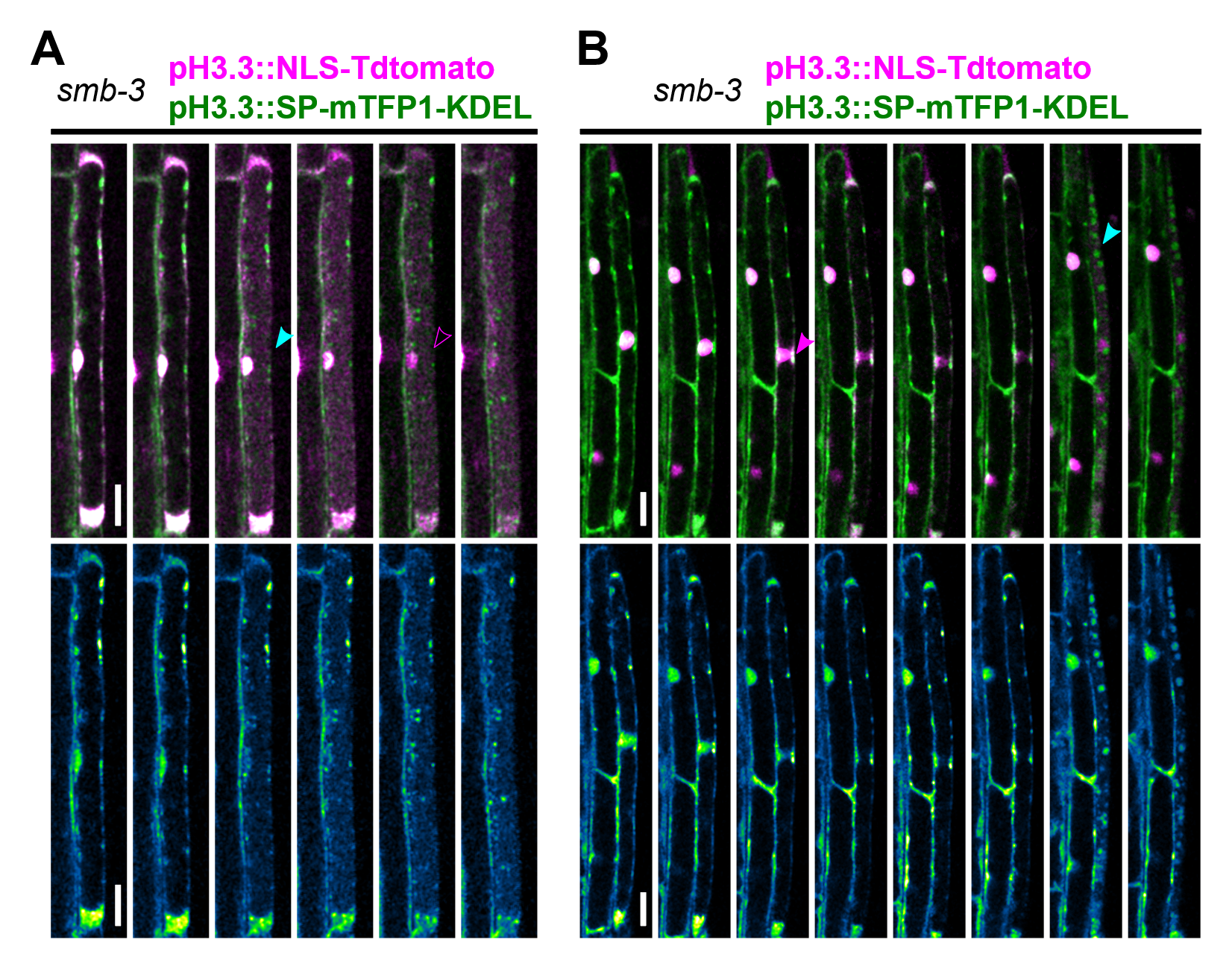


**Supplemental Figure S3: Aberrant dynamics of an ER lumen marker during cell death in the *smb-3* mutant. (A-B)** Two representative confocal time-lapse series of dying LRC cells in 5-day old *smb-3* mutants. Upper panels show NLS-TdTOMATO in magenta and mTFP1 in green, the lower panels show the mTFP1 channel separately. There is no clearly defined release of the ER lumen marker *pH3.3::SP-mTFP1-KDEL* after vacuolar collapse (cyan arrow) and NE breakdown (magenta arrow). Scale bars are 20 μm. **Supports Figure 3**.

**
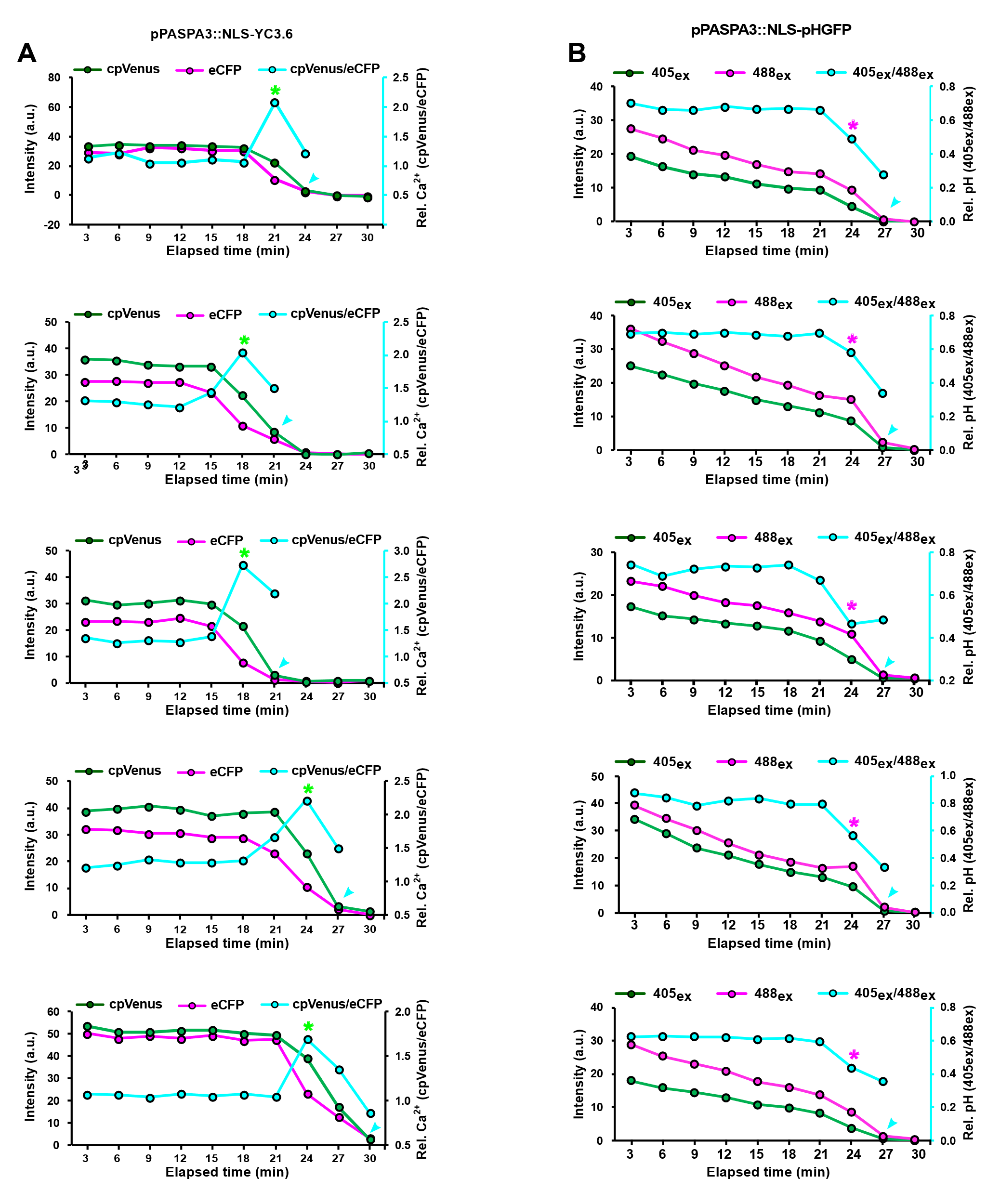
Supplemental Figure S4**

**Supplemental Figure S4: Intracellular calcium transient and acidification precede PCD execution.** Five representative graphs related to figure 4C and D, showing the dynamics and ratios of fluorescent intensities correlating to intracellular [Ca^2+^] **(A)** and pH **(B)** sensors, respectively, expressing in wild-type LRC cells. The ratios of fluorescence intensity were obtained after background noise subtraction for each channel and corresponding to the relative [Ca^2+^]_nuc_ (cpVenus/eCFP) or pH_nuc_ (405ex/488ex). The cyan arrow indicates NE breakdown, the green asterisk in panel A marks the Ca^2+^  transient and the magenta asterisk in panel B marks acidification. **Supports Figure 4**.

**Supplemental Figure S5**

**
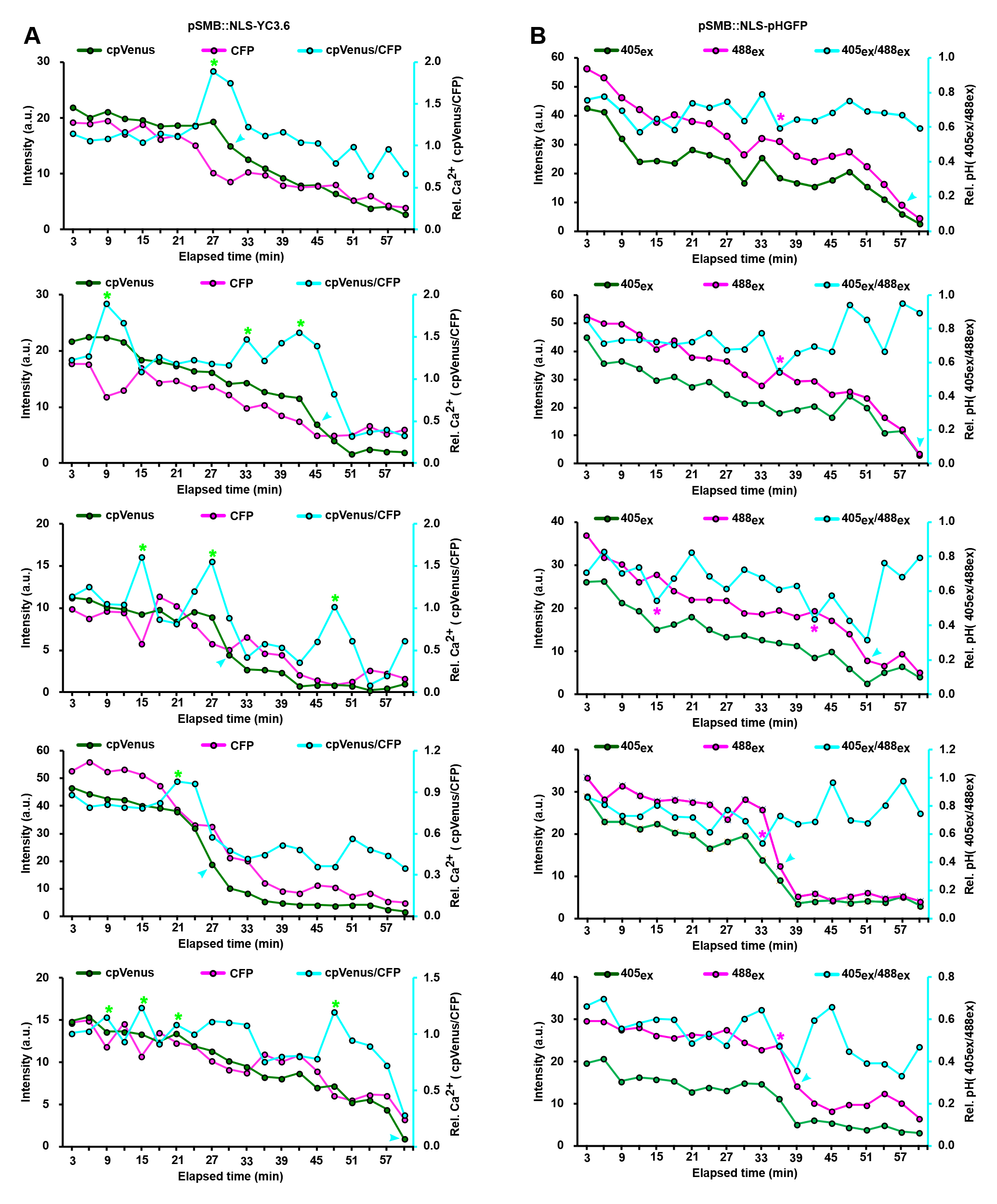
**

**Supplemental Figure S5: Aberrant pattern of intracellular calcium transient and acidification during *smb-3* cell death.** Five representative graphs related to figure 5C and D, showing the dynamics and ratios of fluorescent intensities correlating to intracellular [Ca^2+^] **(A)** and pH **(B)** sensors, respectively, expressing in *smb-3* LRC cells. The ratios of fluorescence intensity were obtained after background noise subtraction for each channel and corresponding to the relative [Ca^2+^]_nuc_ (cpVenus/eCFP) or pH_nuc_ (405ex/488ex). The cyan arrow indicates NE breakdown, the green asterisk in panel A marks the Ca^2+^  transient and the magenta asterisk in panel B marks acidification. **Supports Figure 5**.

**Supplemental Figure S6**

**
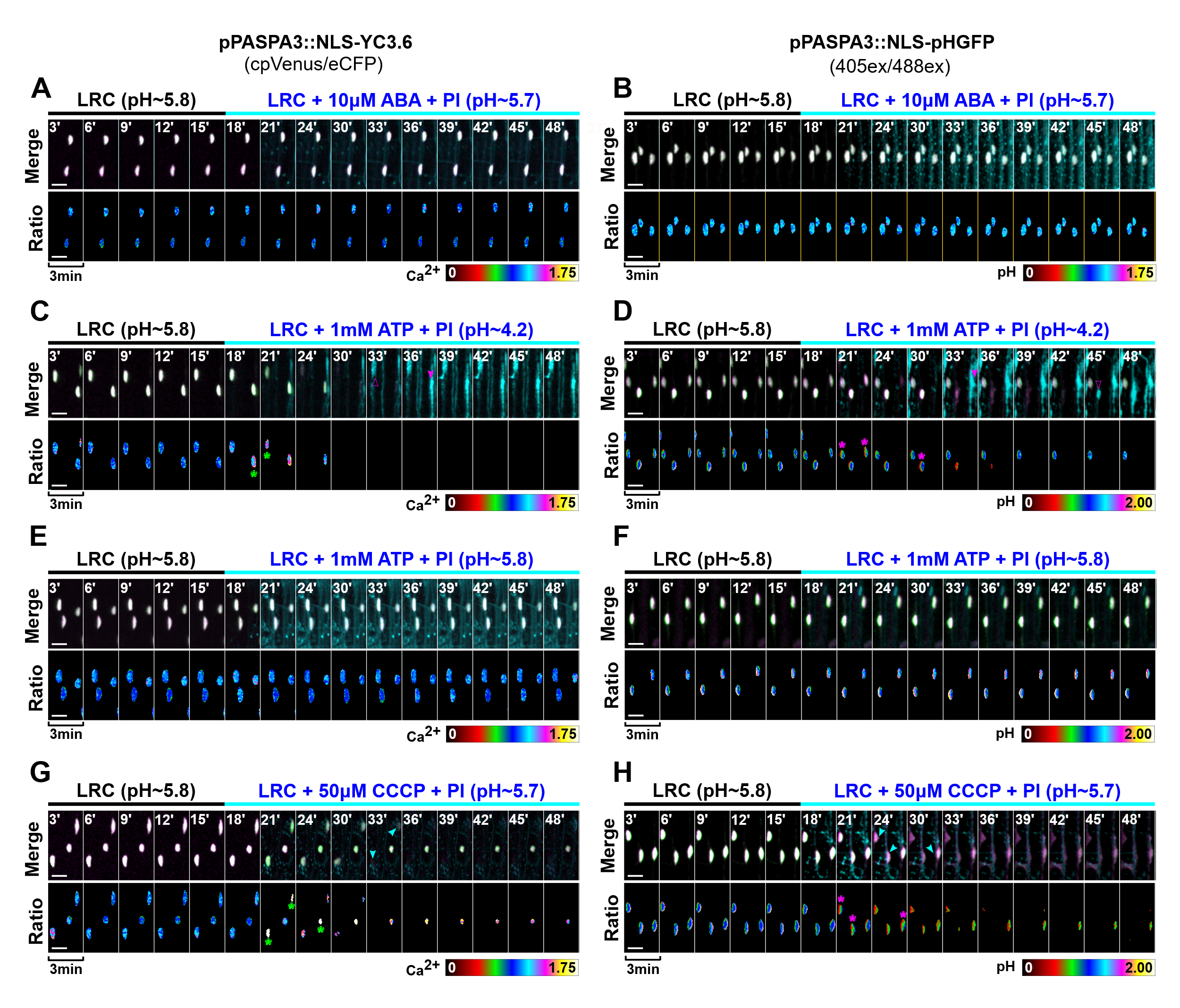
**

**Supplemental Figure S6: Pharmacological treatment causes distinct [Ca^2+^]_nuc_ and pH_nuc_ signatures, which are followed by cell death specifically in distal LRC cells. (A,C,E,G)** Time-lapse confocal images of LRC cells from 4-day-old Col-0 seedlings expressing *pPASPA3::NLS-YC3.6* in response to ABA (n=4 roots), ATP (pH~4.2, n=5 roots; pH~5.8, n=4 roots), and CCCP (n=6 roots) application, respectively. Magenta arrows and cyan arrows indicate PI entry and NE breakdown, respectively, and green asterisks mark the [Ca^2+^]_nuc_ elevation. **(B,D,F,H)** Time-lapse confocal images of LRC cells from 4-day-old Col-0 seedlings expressing *pPASPA3::NLS-NLSpHGFP* in response to ABA (n=7 roots), ATP (pH~4.2, n=4 roots; pH~5.8, n=6 roots) and CCCP (n=6 roots) application, respectively. Magenta arrows and cyan arrows indicate PI entry and NE breakdown respectively, and magenta asterisks mark pH_nuc_ acidification. Upper panels show the merged signals, lower panels show ratiometric imaging. The color schemes are the same as described for Figure 4, cyan indicates the PI signal. Scale bars are 10 μm. **Supports Figure 6**.


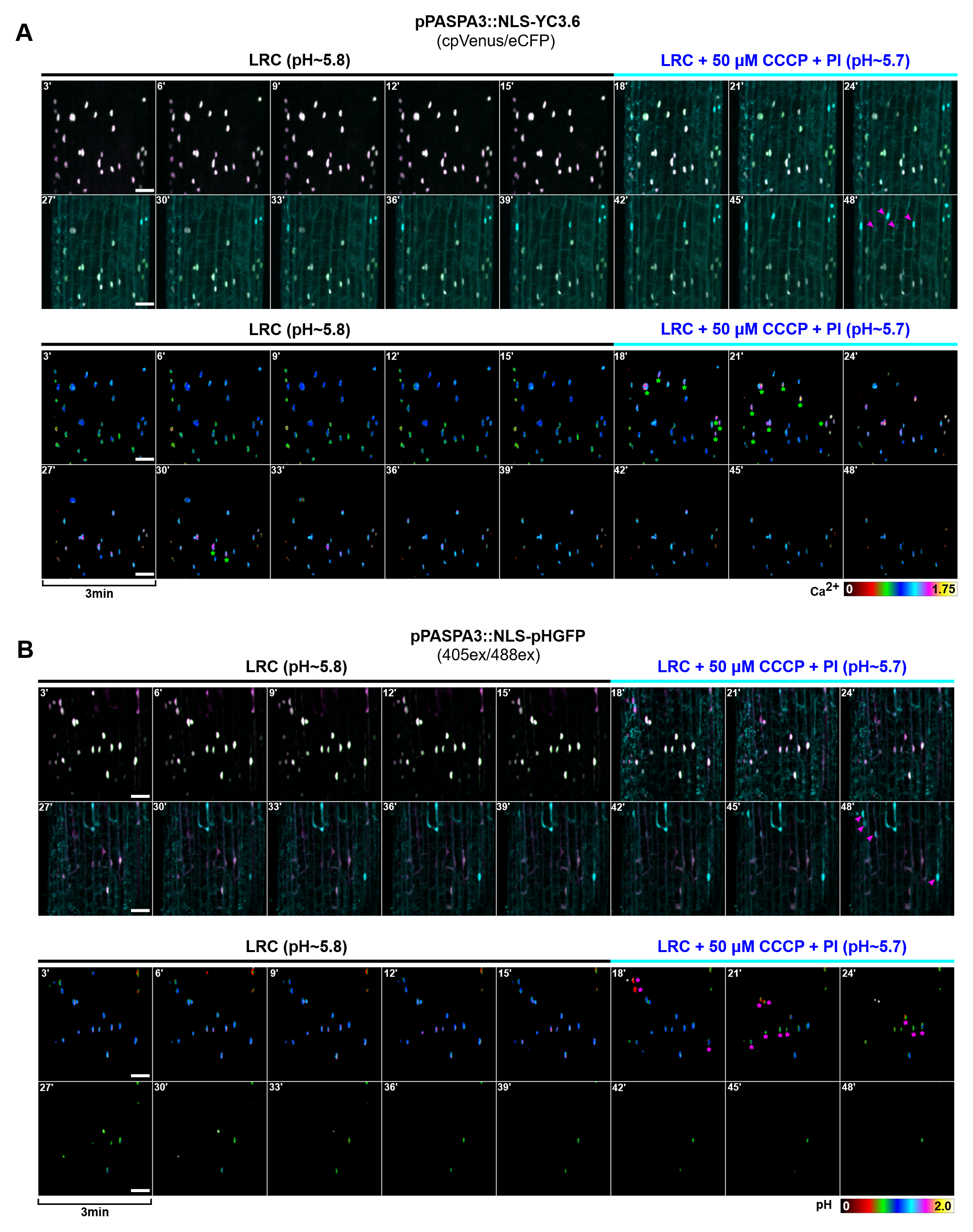
**Supplemental Figure S7**

**Supplemental Figure S7: CCCP treatment causes [Ca^2+^]_nuc_ and pH_nuc_ signatures, which are followed by cell death specifically in distal LRC cells. (A)** Time-lapse confocal images of LRC cells from 4-day-old Col-0 seedlings expressing *pPASPA3::NLS-YC3.6* in response to CCCP application. Magenta arrows indicate PI entry, and green asterisks mark the [Ca^2+^]_nuc_ elevation. **(B)** Time-lapse confocal images of LRC cells from 4-day-old Col-0 seedlings expressing *pPASPA3::NLS-NLSpHGFP* in response to CCCP application. Magenta arrows and cyan arrows indicate PI entry, and magenta asterisks mark pH_nuc_ acidification. Upper panels show the merged signals, lower panels show ratiometric imaging. The color schemes are the same as described for Figure 4, cyan indicates the PI signal. Scale bars are 20 μm. **Supports Figure 6**.

**Supplemental Figure S8**


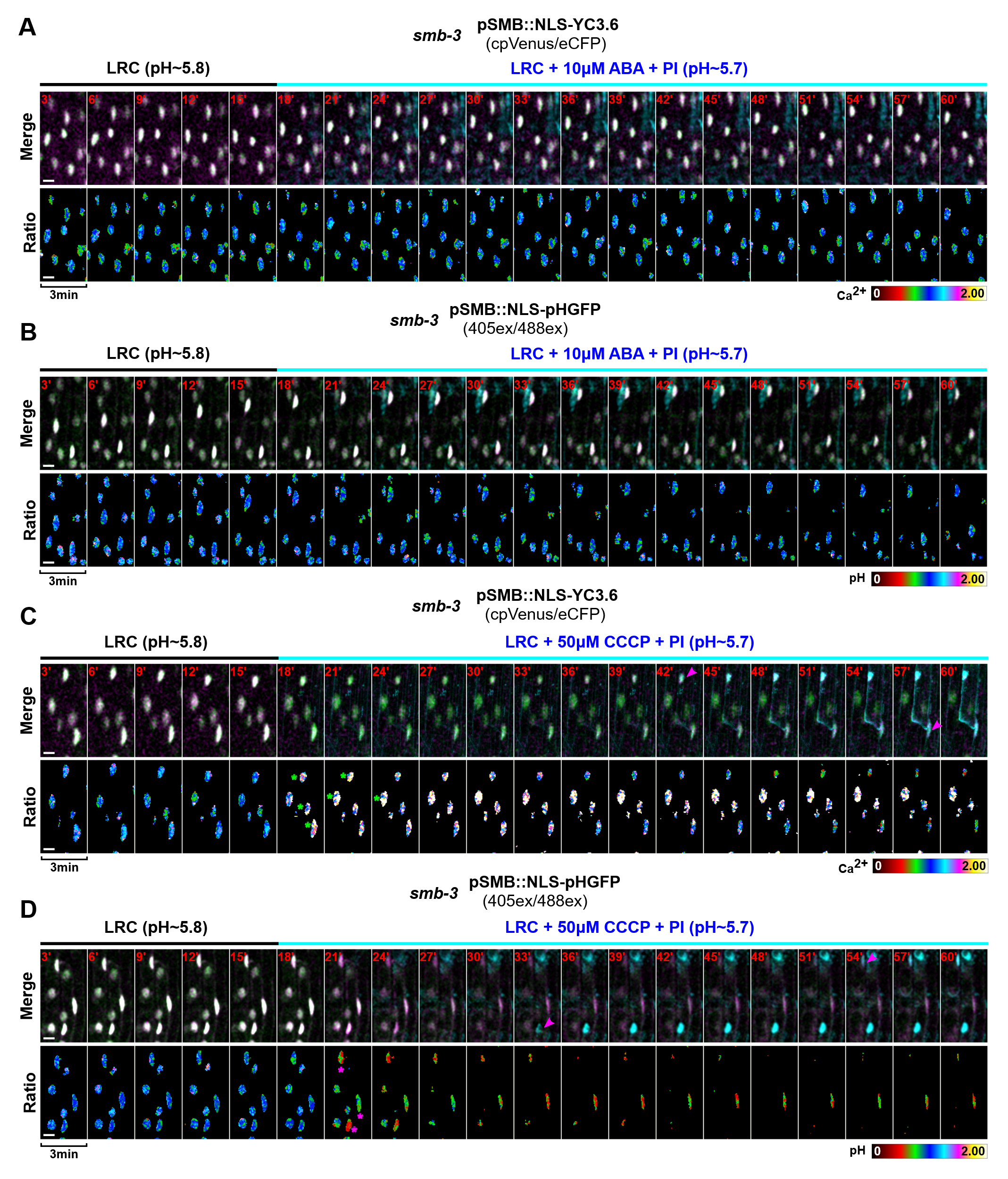


**Supplemental Figure S8: Short-term CCCP treatment induces intracellular calcium elevation and acidification in *smb-3* LRC cells. (A,C)** Time-lapse confocal images of LRC cells from 5-day-old *smb-3* seedlings expressing *pSMB::NLS-YC3.6* showing the response to ABA (n=3 roots) and CCCP (n=7 roots) treatment, respectively. Magenta arrows indicate PI entry, and green asterisks mark the [Ca^2+^]_nuc_ elevation. **(B,D)** Time-lapse confocal images of LRC cells from 5-day-old *smb-3* seedlings expressing *pPASPA3::NLS-pHGFP* showing the response to ABA (n=4 roots) and CCCP (n=8 roots) treatment, respectively. Magenta arrows indicate PI entry, and magenta asterisks mark the pH_nuc_ acidification. Upper panels show the merged signals, lower panels show ratiometric imaging. The color schemes are the same as described for Figure 4, cyan indicates the PI signal.Scale bars are 10 μm. **Supports Figure 7**.

**Supplemental Table S1: Plasmid information**

**Supplemental Table S2: Primer information and sequences**

**Supplemental Table S3: Information on transgenic plant lines used in this study**
