# Supplementary material for: A developmentally controlled cellular decompartmentalization process executes programmed cell death in the Arabidopsis root cap
Source: Plant Cell. Author manuscript; Available in PMC 2024 Sep 29. (PMC7615778; doi:10.1093/plcell/koad308)
Supplement: Movie Legends [file EMS192759-supplement-Movie_Legends.docx]

**Movie Legends:**

**Movie 1: Time-course confocal imaging indicates NE breakdown as reference time point during dPCD execution.** Time-course confocal imaging of a root tip from a 4-day-old Col-0 seedling expressing the nuclear reporter *pSMB::NLS-GFP* stained with PI. The NLS-GFP signal dissipates in the distalmost LRC cell (arrowhead), indicating the rapid occurrence of NE breakdown, followed by PI entry into the cell (magenta signal). A maximal Z-projection of 3 slices is shown. Root growth was compensated for by registration. **Supports Figure 1.**

**Movie 2: Time-course confocal imaging indicates NE breakdown occurs concurrently with ER leakage during dPCD.** Time-course confocal imaging of a root tip from a 4-day-old Col-0 seedling expressing *pPASPA3::NLS-TdTOMATO*and *pTATD::SP-mTFP1-KDEL* dual-color markers. In the dying cells of the distalmostdistalmost LRC cells, NE breakdown is accompanied by ER leakage into the cytosol, preceding vacuolar collapse indicated by even distribution of the fluorescent signal in the entire cell volume. A maximal Z-projection is shown. Root growth was compensated for by registration. **Supports Figure 1.**

**Movie 3: Time-course confocal imaging indicates mitochondrial matrix leakage prior to NE breakdown during dPCD.** Time-course confocal imaging of a root tip from a 4-day-old Col-0 seedling expressing *pPASPA3::NLS-TdTOMATO*and *pTATD::COX4-mTFP1* dual-color markers. In the dying cells of the distalmost LRC cells, mitochondria leakage, indicated by abrupt disappearance of mitochondrial foci, occurs prior to NE breakdown. A maximal Z-projection is shown. Root growth was compensated for by registration. **Supports Figure 1.**

**Movie 4: Time-course confocal imaging indicates NE breakdown occurs concurrently with PM endodomain shedding during dPCD.** Time-course confocal imaging of a root tip from a 4-day-old Col‑0 seedling expressing *pPASPA3::NLS-TdTOMATO and pPASPA::CPK17-mTFP1* dual-color markers. During dPCD in the distalmost LRC cells, NE breakdown occurs simultaneously with PM endodomain shedding (indicated by strong cytoplasmic signal), and prior to vacuolar collapse (indicated by an even distribution of the fluorescent protein in the entire cell volume). A maximal Z-projection is shown. Root growth was compensated for by registration. **Supports Figure 2.**

**Movie 5: Time-course confocal imaging indicates generation of extracellular vesicles during dPCD.** Time-course confocal imaging of a root tip from a 4-day-old Col-0 seedling expressing *pPASPA3::ToIM*. During dPCD occurring in the distalmost LRC cells, extracellular vesicles can be seen blebbing out of degrading cell corpses on the root surface. Note that living cells show a strong cytoplasmic GFP signal (green) and degrading cell corpses show an evenly distributed RFP signal (magenta). A maximal Z-projection is shown. Root growth was compensated for by registration. **Supports Figure 2.**

**Movie 6: Time-course confocal imaging indicates gradual dissipation of nuclear localized fluorescent proteins during cell death in the *smb-3* mutant.** Time-course confocal imaging of a root tip from a 5-day-old *smb-3* seedling expressing the nuclear reporter *pSMB::NLS-GFP* stained with PI. In the dying LRC cell (nucleus indicated by arrowhead), the NLS-GFP signal disappears more gradually than in the wild type, followed by PI entry. A maximal Z-projection is shown. Root growth was compensated for by registration. **Supports Figure 3.**

**Movie 7: Time-course confocal imaging indicates delayed mitochondrial matrix release during cell death in the *smb-3* mutant.** Time-course confocal imaging of a root tip from a 5-day-old *smb-3* seedling expressing *pH3.3::NLS-TdTOMATO*and *pH3.3::COX4-mTFP1* dual-color markers. In the dying LRC cells, mitochondrial foci remain visible after NE breakdown and vacuolar collapse. A maximal Z-projection is shown. Root growth was compensated for by registration. **Supports Figure 3.**

**Movie 8: Time-course confocal imaging indicates the aberrant PM endodomain shedding during cell death in the *smb-3* mutant.** Time-course confocal imaging of a root tip from a 5-day-old *smb-3* seedling expressing *pH3.3::NLS-TdTOMATO*and *pH3.3::CPK17-mTFP1* dual-color markers. In the dying LRC cells, cytosolic solubilization of CPK17-mTFP1 is only completed after NE breakdown in *smb-3* mutants. A maximal Z-projection is shown. Root growth was compensated for by registration. **Supports Figure 3.**

**Movie 9: Time-course confocal imaging indicates the intracellular calcium transient during dPCD.** Time-course confocal images (left) and false-color ratio images (right) from a 4-day-old Col-0 seedling expressing the calcium sensor *pPASPA3::NLS-YC3.6*. In the dying distal LRC cells, a sharp transient increase of Ca^2+^ ions in the nucleoplasm occurs prior to NE breakdown. A maximal Z-projection of 3 slices is shown. Root growth was compensated for by registration. **Supports Figure 4.**

**Movie 10: Time-course confocal imaging indicates the intracellular acidification during dPCD.** Time-course confocal images (left) and false-color ratio images (right) from a 4-day-old Col-0 seedling expressing the pH sensor *pPASPA3::NLS-pHGFP*. In the dying distal LRC cells, acidification of the nucleoplasm occurs prior to NE breakdown. A maximal Z-projection of 3 slices is shown. Root growth was compensated for by registration. **Supports Figure 4.**

**Movie 11: Time-course confocal imaging indicates the aberrant patterns of intracellular calcium transient during *smb-3* cell death.** Time-course confocal images (left) and false-color ratio images (right) from a 5-day-old *smb-3* seedling expressing a calcium sensor *pSMB::NLS-YC3.6*. In the dying distalmost LRC cells, an increase of Ca^2+^ ions in the nucleoplasm is followed by a gradual occurrence of NE breakdown. A maximal Z-projection from 3 slices is shown. Root growth was compensated for by registration. **Supports Figure 5.**

**Movie 12: Time-course confocal imaging indicates the aberrant patterns of intracellular calcium transient during *smb-3* cell death.** Time-course confocal images (left) and false-color ratio images (right) from a 5-day-old *smb-3* seedling expressing a pH sensor *pSMB::NLS-pHGFP*. In the dying distalmost LRC cells, a slow and gradual acidification occur before NE breakdown (cyan arrow). A maximal Z-projection of 3 slices is shown. Root growth was compensated for by registration. **Supports Figure 5.**
